# Guidelines for the Measurement of Rein Tension in Equestrian Sport

**DOI:** 10.3390/ani11102875

**Published:** 2021-09-30

**Authors:** Hilary Clayton, Russell MacKechnie-Guire, Anna Byström, Sarah Le Jeune, Agneta Egenvall

**Affiliations:** 1 Department of Large Animal Clinical Sciences, Faculty of Veterinary Medicine, Michigan State University, 736 Wilson Road, East Lansing, MI 48824, USA; claytonh@msu.edu; 2 Centaur Biomechanics, Moreton Morrell, Warwickshire CV35 9BB, UK; russell@centaurbiomechanics.co.uk; 3 Department of Anatomy, Physiology and Biochemistry, Faculty of Veterinary Medicine and Animal Science, Swedish University of Agricultural Sciences, P.O. Box 7046, SE-750 07 Uppsala, Sweden; Anna.Bystrom@slu.se; 4 Department of Surgical and Radiological Sciences, University of California, Davis, CA 95616, USA; sslejeune@ucdavis.edu; 5 Department of Clinical Sciences, Faculty of Veterinary Medicine and Animal Science, Swedish University of Agricultural Sciences, P.O. Box 7054, SE-750 07 Uppsala, Sweden

**Keywords:** rein sensor, horse, rider, calibration, analysis, magnitude, peak

## Abstract

**Simple Summary:**

The reins are used to control speed and direction of the horse’s movement through the application of tension by the rider. When the rider holds the reins with a constant light contact, the mechanics of each gait is associated with a cyclic pattern of head and neck movements that is revealed in rein tension oscillations that have a typical shape and repetition frequency in each gait. The effects of the rider’s aids, rider imbalance and extraneous movements of the horse’s head and neck are superimposed on the basic patterns of the gaits. Rein tension is of interest to scientists and horsemen alike. Tension is relatively easy to measure but the equipment, analytic techniques and reporting of rein tension vary greatly. This paper makes recommendations to guide the selection of suitable equipment and appropriate methods for the collection, analysis and reporting of rein tension data. The goals are to describe correct procedures and common pitfalls in the collection, analysis and reporting of rein tension data that will facilitate comparisons between different studies.

**Abstract:**

Rein tension is relatively easy to measure, and the resulting data are useful for evaluating the interaction between horse and rider. To date, there have been a number of studies using different transducers, calibration methods and analytical techniques. The purpose of this paper is to make recommendations regarding the collection, analysis and reporting of rein tension data. The goal is to assist users in selecting appropriate equipment, choosing verified methods of calibration, data collection and analysis, and reporting their results consistently to facilitate comparisons between different studies. Sensors should have a suitable range and resolution together with a fast enough dynamic response, according to the gait, speed and type of riding for which they will be used. An appropriate calibration procedure is necessary before each recording session. A recording frequency of 50 Hz is adequate for most rein tension studies. The data may be analyzed using time-series methods or by extracting and analyzing discrete variables chosen in accordance with the study objectives. Consistent reporting facilitates comparisons between studies.

## 1. Introduction

Equestrianism is based on a system of communication between rider and horse in which the horse learns to respond appropriately to cues or ‘aids’ from the rider. In the age of microelectronics and wearable sensors, researchers have developed methods aimed to measure the riders’ cues, their effects on the horse, and the horse’s responses. The availability of cost effective and user-friendly measuring devices has facilitated transitioning the use of these tools from the research lab to the field, which is one of the goals of equestrian research [1,2]. The use of technology in the field, however, depends not only on having accurate, reliable, and robust systems for data acquisition, but also on analysing the data and interpreting the results correctly. 

Measuring rein tension is a relatively simple process requiring a transducer between the rein and bit or within the rein, a power source, and a means of exporting the data to suitable storage and display devices. Several turnkey systems for measuring rein tension have become available commercially. In spite of the apparent simplicity of the process of measuring rein tension, a number of pitfalls await the unwary. This paper is not intended to be a review of the rein tension literature (see [1] for a recent summary) but rather, it is a ‘how to’ guide to the measurement and analysis of rein tension data, with citations being limited to those that illustrate specific points.

Studies of rein tension appeared in the scientific literature around the turn of the twenty-first century. Preuschoft et al. (1999) used a single strain gauge transducer attached to one rein. The sensor, together with its battery and circuitry, weighed 300 g [3]. It was so heavy that only one rein could be instrumented at a time. This limitation highlighted the importance of using lightweight components to limit the weight of equipment attached to the rein and the horse’s head. Clayton et al. used sensors weighing 21 g and 85 g together with attachment hardware weighing approximately 75 g. This system provided accurate measurements up to 1334 N, to explore the magnitude and pattern of rein tension [4,5]. These studies concluded that sensors used for rein tension measurement when riding on the flat should have a range >200 N to allow a margin of safety against over-loading the sensor [4]. The authors suggested that their methodology could be improved by having sensors in both reins to measure left-right coordination and symmetry patterns, a telemetry system to dispense with the need for a cable attaching the horse to the computer, a means of synchronizing tension data with kinematic data to correlate the timing of rein tension with stride events, and a method of providing immediate feedback to the rider.

Following these pioneering works, many more rein tension studies have been published and rein tension transducers have undergone significant technical development and miniaturization. A review of rein tension research [1] highlighted a diversity in instrumentation, data management and reporting. The objective of this paper is to provide guidance on the measurement, analysis, interpretation, and reporting of rein tension data in an effort to harmonize standards for collecting and disseminating these types of data.

## 2. Basic Characteristics of the Rein Tension Signal

When initiating research in a novel area, such as rein tension evaluation, the first step is to gain a basic understanding of the magnitude, frequency, and pattern of the signal that will be measured. This information is necessary to select appropriate measuring equipment, to have it calibrated to the appropriate range, and to determine the necessary sampling frequency.

Rein tension measures a pulling force applied to the rein by the horse and/or rider. Tension values alone cannot distinguish between these two sources. Each gait has a characteristic rein tension pattern produced by mechanical oscillations of the horse’s head and neck repeated on a stride-by-stride basis [6]. This gives rise to a repetitive pattern of spikes that occur at fairly regular intervals with intervening decreases to a minimum or baseline value. Superimposed on this basic pattern are the effects of the rider’s aids, which can differ in magnitude, duration, and timing within the stride and so add a level of unpredictability. Furthermore, the impossibility for the rider to be perfectly coordinated with the horse’s motion, together with extraneous movements of the horse’s head, contribute to variability in the signal.

**Figure 1:**
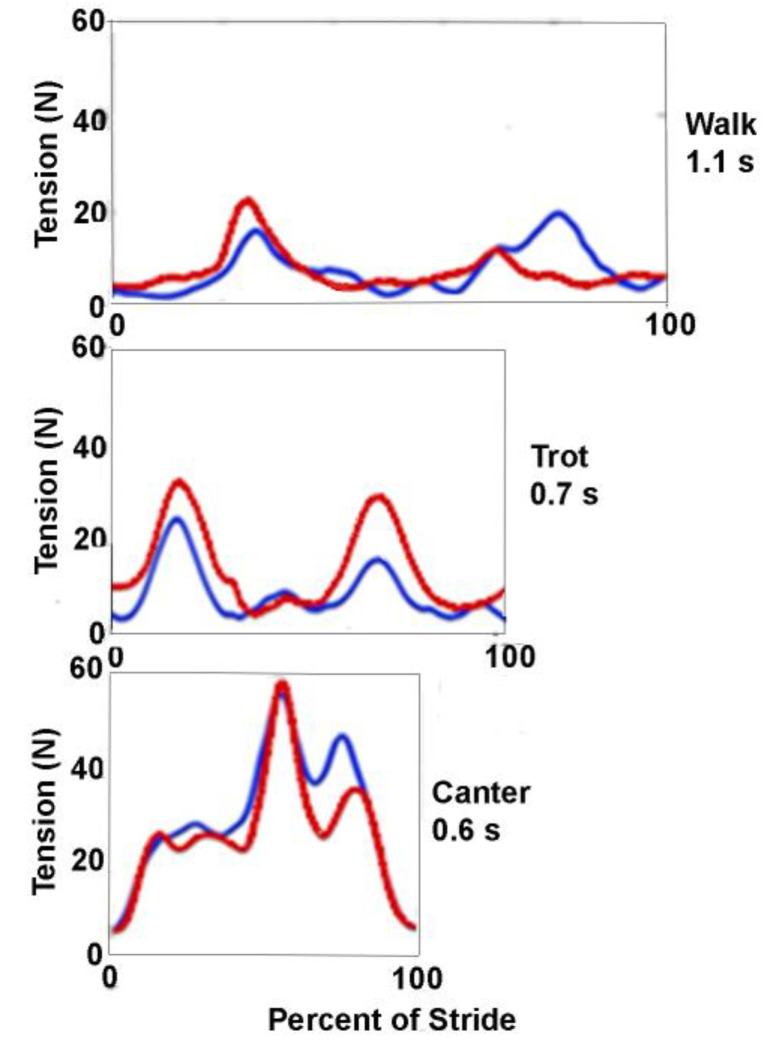
Rein tension traces with a rider for left rein (blue) and right rein (red) during one stride of walk (upper graph), trot (middle graph) and canter (lower graph). All graphs shown at the same tension scale on y axis. The x axis of each graph is labelled as percent of stride (i.e., time normalized) with the actual width of each graph corresponding to its stride duration which is shown top right below the name of the gait.

In walk there are two rein tension spikes per stride cycle. These relate to the obvious nodding motion of the head and neck, when the croup oscillates, which is a component of the inverted pendulum dynamics of the gait that is a mechanism for reducing energy expenditure [7]. Riders try to maintain a relatively constant rein tension by allowing their hands to follow the motion of the horse’s head. This, in combination with the walk having no suspension phases, results in the rein tension spikes being small in magnitude (Figure 1). The two spikes within each stride are, however, often different in magnitude. A study using side reins reported a single large spike in the rein ipsilateral to the forelimb as it leaves the ground coordinated with a smaller spike in the contralateral rein [8]. The graph shown in Figure 1 has a higher spike in the rein ipsilateral to the forelimb leaving the ground and a smaller spike in the contralateral rein. This may relate to the fact that walking horses display some lateral movement of the head between left and right forelimb stance phases [8] and that when side reins are used, there is no compensation for asynchronous changes in tension as there might be with a rider.

In trot, the horse’s body undergoes two vertical excursions per stride; the body is highest in the transitions between diagonal stance phases which coincide with the minima in rein tension. The body reaches its lowest point in the middle of diagonal stance, after which the head and neck continue their descent until the nuchal ligament and dorsal cervical musculature restrain the downward motion and raise the neck. Rein tension increases as the head descends relative to the base of the neck and maximal tension coincides with the lowest point of the head, which occurs during the second half of each diagonal stance phase [6,8,9] (Figure 1). Coordinated movements of the rider’s shoulder and elbow joints allow the wrist to maintain a consistent position relative to the bit, changing by <15 mm during each stride [10] as the rider’s hands follow the bit.

In canter the horse undergoes a single suspension in each stride with a corresponding vertical oscillation; the body is highest in the suspension phase and lowest when the synchronized diagonal limbs are at midstance. In addition to the vertical oscillation, the horse shows a marked pitching rotation in each stride. Nose up pitch is maximal in suspension and nose down pitch is maximal in the stance phase around the time when the body reaches its lowest point [11]. The rein tension shows a large peak that occurs close to the time of the lowest point in the stride [12,13] coinciding with the lowering of the head and neck. Smaller peaks are often present before and after the major peak, representing the action of the individual limbs [13] (Figure 1).

When a rider plans a transition from walk to trot or canter, the reins are usually shortened to maintain a consistent contact with the bit. The rider’s shoulder and elbow flex and extend in synchrony with the pitching motion of the rider’s trunk to maintain the hand at a fairly consistent distance from the bit [10]. If side reins are used for comparison with a rider, their length should be adjusted for each gait to equate with the rider’s adjustments in rein length. For example, compared with the neutral length of the horse’s neck, side reins need to be shortened by about 10 cm to be equivalent to the rider’s contact in trot [6].

## 3. Technical Requirements for a Rein Tension Measurement System

This section describes the technical requirements for rein tension transducers and their calibration, options for data display and storage, and integration of the components into a system for measuring rein tension. Recommendations are based on published data —sources of which can be seen in the reference list.

### 3.1. Sensor Type

The ideal transducer for measuring tensile strain within the rein or between the bit and the rein should be lightweight, accurate and reliable throughout the range of measured tensions and should be sufficiently robust to withstand equestrian activities. Strain gauge transducers and piezoelectric load cells meet these requirements and are the sensor types most commonly used in research. 

**Figure 2:**
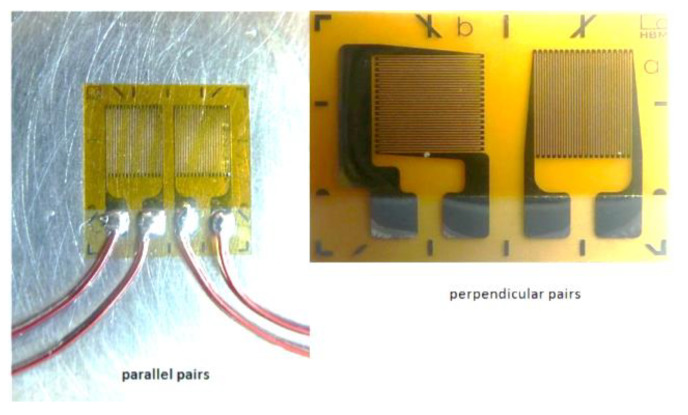
Examples of strain gauges. Left: the metallic foil pattern, is arranged in parallel; right: the metallic foil patterns of the two strain gauges are arranged perpendicular to each other (image from [14].

A strain gauge transducer is based on a fine wire or metallic foil electrical conductor arranged in a grid pattern (Figure 2). When tension is applied, the grid is stretched, becoming longer and narrower, which increases its electrical resistance and changes the voltage in the output leads. This analogue signal is then converted to digital values by an A-D (analogue to digital) converter. The digital values are then displayed, captured and/or analyzed. A downside of strain gauges is that they are temperature sensitive, but manufacturing modifications can reduce drift due to changes in ambient temperature (i.e., make them temperature-compensated). A benefit is that they can measure both static and dynamic loads.

Piezoelectric load cells are based on the use of quartz crystals that generate an electrostatic charge when force is applied to or removed from them. The output force is proportional to the input force. These sensors only generate an output under dynamic loads and cannot be used for static measurements. They are particularly effective for fast measurements of small forces and, since constant non-zero tension seldom occurs during riding, they are suitable for use in rein sensors. Piezoelectric load cells are more expensive than strain gauge transducers but advantages are that they provide greater precision, are less likely to drift and are less influenced by temperature. 

### 3.2. Range

Range is the span between the maximum and minimum values a transducer can measure. The upper limit of the sensor range represents the maximum strain that can be recorded accurately and without damage to the sensor. The lower limit should be zero. The necessary range depends on the gait, speed and type of activity under which measurements will be taken. For sport horses performing on the flat, a range of at least 200 N was recommended in an early study [5] but subsequent studies have indicated this is inadequate [15]. Furthermore, if the horse performs a behavior such as rooting on the reins, considerably higher forces may be applied. Sports that involve jumping or racing, likely also generate higher values.

**Figure 3:**
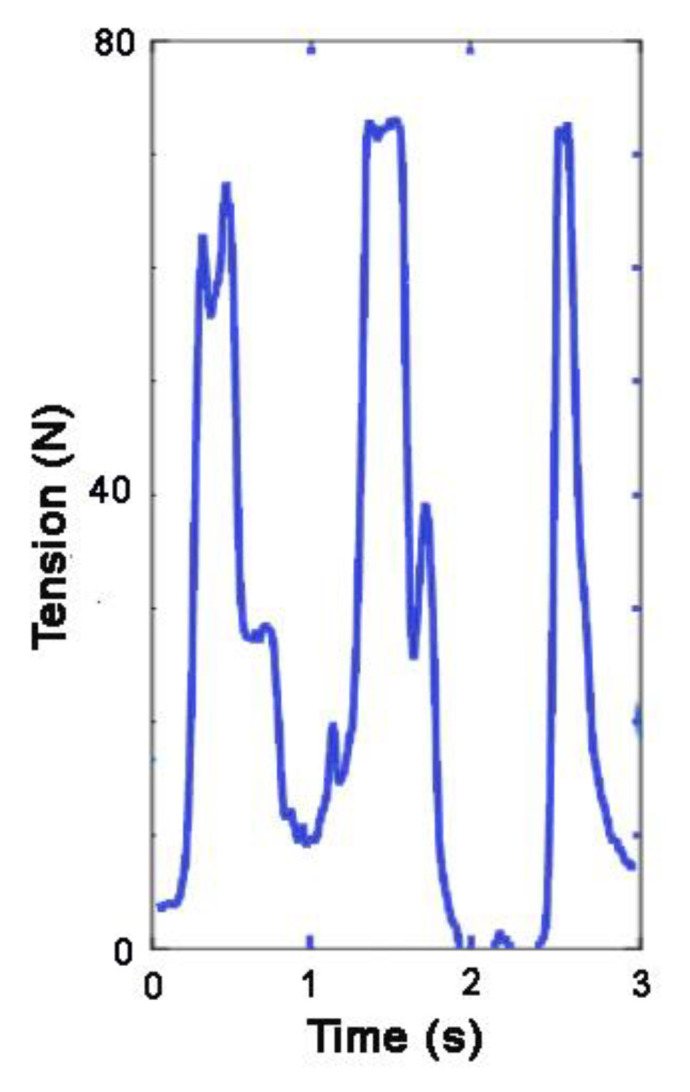
Example of a trace in which the maximal value of the sensor was exceeded for the two spikes on the right.

Selecting a sensor with an adequate range is critical for accurate data collection because when the sensor’s range is inadequate, the tension trace may show a flat line instead of rising to a distinct maximal value (Figure 3). The flat line would then represent an underestimation of the applied tension, which leads to problems in interpretation of the data. Consequently, the mean value would also be underestimated, making true quantification of rein tension relative to the condition evaluated or the gait challenging. However, median values could still be reliable, provided that <50% of the values were above the upper limit of the sensor’s range. In the near future, rein tension measuring equipment using interchangeable sensors with varying ranges would overcome this limitation.

For example, using a load cell with an upper limit of 49 N, ~10% of the data would be above the sensor threshold during a four-minute dressage test [16] and ~15% of data would exceed the sensor threshold during a ten-minute dressage test [17]. Given that dressage tests usually contain walk, trot and canter, with canter having the highest tension, the data loss would be most severe in canter. 

Because minimal rein tension is expected to drop to zero at times, the sensor also needs to measure low tension values accurately. The minimum stride-based rein tension values in ridden walk were found to have a median value <5 N among 8 riders and the minima were greater than zero [15].

### 3.3. Sensitivity and Resolution 

Sensitivity is the ratio between the physical input signal and the electrical output signal.

Resolution is the smallest increment an instrument can detect and display the signal.

Sensitivity is expressed as the change in output of the sensor per unit change in input value. In practice, the A-D converter (typically 2, 4, 8, or 16-bit) is usually the limiting factor. An A-D converter with 8-bit resolution limits the output to 256 unique values and if the range of the sensor is 200 N, then the smallest increment that will be detected is 200/256, i.e., 0.78 N. The digital output thus gives a rather crude representation of the signal. A 16-bit system is, therefore, preferable for most purposes. 

### 3.4. Dynamic Response

Dynamic response describes how rapidly the transducer reacts to a change in applied force. The dynamic characteristics represent how long it takes between the application of a certain level of tension and the sensor returning this as a voltage output. If the dynamic response time is too long, short-lived peaks in the data will be missed because the sensor fails to reach full output. Additionally, baseline or minimal tension will be overestimated because if the rein tension drops rapidly the sensor output drops towards zero more slowly. Measurements affected by the dynamic response of the sensor include rise time, fall time, and peak times. 

Slow dynamic response has been described as a source of error in rein tension measurements. One study used a spring-based system to measure rein tension. The spring had a maximum extension of 15 mm with a measurement range of 0–39 N [18]. Although the use of a spring was described as a cost-effective approach, the slow dynamic response rendered it unsuitable for the purpose of measuring rein tension.

### 3.5. Sampling Frequency

Sampling frequency indicates the number of samples taken from a continuous signal per unit of time. It is measured in hertz (Hz) or samples per second.

**Figure 4:**
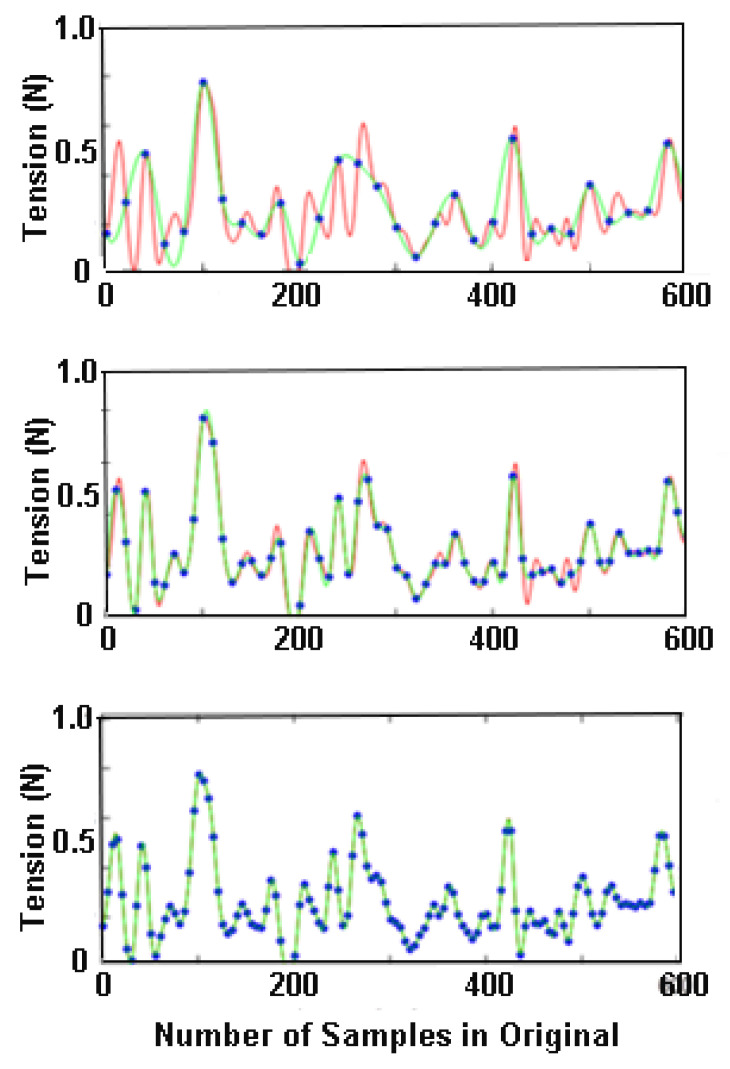
Rein tension curve from a horse trotting with loose reins originally sampled at 240 Hz (red lines). The blue dots represent the data points for each sampling rate (12, 24, 48 Hz) and the green lines are the reconstructed curves after down-sampling to 12 Hz (upper graph), 24 Hz (middle graph), and 48 Hz (lower graph) and then up-sampling to the original sampling rate. The signal is not well preserved after down-sampling to 12 Hz. At 24 Hz some small peaks and troughs are missed and the values of large peaks tend to be under-estimated. At 48 Hz most features of the original 240 Hz curve are preserved.

Rein tension varies continuously over time. Tension values are recorded at regular intervals and the resulting series of data points are used to construct a curve representing the original signal. Accurate reproduction of the original signal requires a sufficiently high sampling frequency to capture the events of interest. According to the sampling theorem, the minimal sampling rate should be at least twice as high as the highest frequency within the time series signal. This is true even if the highest frequencies are not of interest, since high frequency noise may cause low frequency distortion (aliasing). A medium-speed trot has approximately 1.5 strides or 3 diagonal steps per second (Figure 1) and rein tension oscillates at the step frequency, i.e., 3 Hz. The sampling theorem suggests using a minimal sampling frequency of 6 Hz. However, experience has shown that this is insufficient to reproduce the curve accurately and will inevitably miss some events of interest (Figure 4). One of the reasons for needing a higher sampling frequency is that rein tension events are not solely dependent on the horse’s gait cycle; rider-horse interactions produce additional peaks in the data that require higher sampling frequencies. The same has also been described for measuring the vertical oscillation of the axial body segments, which also have a frequency of around 3 Hz at trot [19]. These authors showed that for an inertial sensor mounted on the croup, root mean square errors at specific sampling frequencies were <2 mm at 50 Hz, <4 mm at 40 Hz, <7 mm at 25 and 35 Hz, and 20 mm at 20 Hz and below. Thus, reducing the sampling frequency to 50 Hz had minimal effect on the measured data in that study.

With regard to rein tension data, a sampling frequency of 100 Hz is likely adequate for most studies though a minimum sampling frequency of 50 Hz seems appropriate for most riding activities. The lower limit for recording rein tension data has not been established, but there are some data to show how interpretation of the data are limited by a sampling frequency that is too low. Figure 4 shows the result of down-sampling a signal recorded at 240 Hz to 12, 24 and 48 Hz. At 12 and 24 Hz small spikes are missed or under-estimated whereas at 48 Hz most of the original features are preserved. Veen et al. (2018) recorded rein tension data at 10 Hz in walk, in which rein tension spikes have a frequency <2/sec [20]. The raw data were under-sampled which made it challenging to follow fluctuations of the rein tension signal in detail. 

Higher sampling frequencies may be needed when the application of sudden rein aids or rapid fluctuations in tension can be expected, as might occur during jumping or racing. If interrogating the patterns within short intervals such as changes in rein tension during transitions or flying changes, then a higher sampling frequency may also be warranted.

### 3.6. Attachment of the Transducer

**Figure 5:**
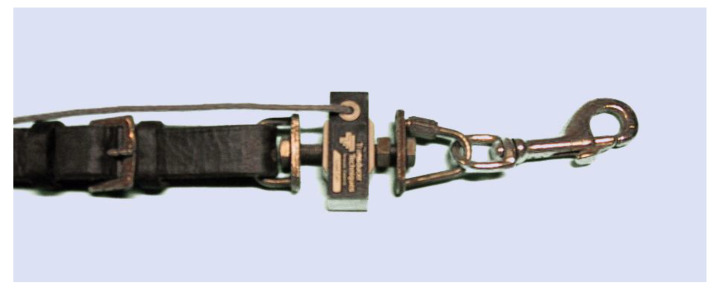
Strain gauge attached securely to the rein (left) with a metal clip for attachment to the bit. Note that none of the components are elastic. (image from [5]).

Transducers can be embedded into a custom designed rein or attached between the end of the rein and the bit rings where lightweight clips allow rapid attachment. The attachments must be sufficiently durable to avoid breakage and the inherent safety risks. It is important to not have any elastic elements in the attachment hardware and sensors (Figure 5). It is also important that attachments are lightweight, since their weight applies gravitational and inertial forces to the rein and the bit and affects the rider’s feel.

### 3.7. Calibration 

Calibration compares measurements made by a sensor with a reference standard of a known value. Calibration is an important process to ensure accuracy and consistency of the data over time and across different measurement devices. Although strain gauge and piezoelectric transducers are known for their stability and reliability, they should be calibrated before and, if necessary, during or after each study. The linearity of the relationship between the raw voltage signal and rein tension should be evaluated, to determine the value in the raw signal that corresponds to 0 N. Even after offset correction, small negative values in the measured signal may have to be adjusted to zero during post-processing. When calibrating a proprietary rein tension measurement system, a difference of 0.1 kg between left and right sensors was reported [21]. A difference of this magnitude would produce a systematic error in the data thus emphasising the need for calibration.

**Figure 6:**
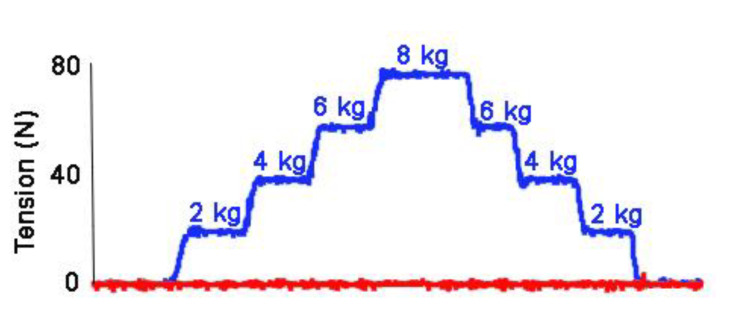
Calibration of a transducer by hanging weights of 2, 4, 6, 8, 6, 4, and 2 kg from the transducer and recording the output voltage which is converted to Newtons. In this example weights were added to the right rein at intervals of 10 s and removed at intervals of 6–8 s, Red: right rein; blue: left rein.

The preferred calibration method is to apply a series of known weights that cover the entire range of values to be measured and record the voltage changes. The weights should preferably be applied in both ascending and descending sequences (Figure 6) [4]. The regression calculation from the calibration indicates the accuracy and linearity of the system.

In practice, the applied or recommended calibration procedures vary. One commercially available system requires the rein tension device to be placed on a horizontal surface with no applied load to set the system to ‘zero’. This procedure simply sets the unloaded sensors to zero but does not evaluate the accuracy or linearity of the output over a range of values. Another system recommends suspending a single known weight from the transducer which indicates accuracy at a single data point only and does not evaluate the output signal over a range of applied tensions. A more thorough calibration as described above can be performed with most systems. Calibration procedures should be detailed in all manuscripts describing rein tension measurements. A recent review found that calibration procedures were reported in twelve of seventeen studies [1].

### 3.8. Reporting Technical Specifications 

When choosing the optimal sensor, there is often a tradeoff between range, resolution (precision), and cost. All of these should be factored into the purchasing decision. When acquiring a turnkey system, manufacturers may be unwilling to provide full system specifications [1]. Consequently, researchers may end up using equipment for which they do not know the full technical specifications [1]. Some labs put together custom-built systems in which all components are chosen to fit the researchers’ specifications, which is an ideal solution when the necessary technical skills are available.

In general, it is recommended that technical specifications be reported to facilitate comparisons between studies and to inform the design of future studies. When the full specifications are not available to the researcher, the name of the system and manufacturer should be given together with any available information. Manufacturers are encouraged to share this information with researchers. 

### 3.9. Data Storage

During measurements, data can be stored locally, for example using a data storage unit attached to the bridle or on a smart phone carried by the rider and later downloaded to a computer. Alternatively, data can be transmitted wirelessly via Bluetooth to a computer or smartphone, or both. Note that when transmitting wirelessly via Bluetooth the signal range may be a limiting factor. When sensors are taken in and out of range from the receiver, the data strings will be compromised resulting in gaps. Simultaneous local storage of data can then protect against lost data packages. Users need to be aware of how the system manages data gaps; if it automatically smooths the data to remove variability when compensating for gaps, it can have a significant effect on the values recorded and the subsequent analyses. 

### 3.10. Data Display

For sensor systems that support wireless data transmission, the rein tension signal can be viewed in real time on a computer. This approach is useful for checking the quality of the signal and for starting/stopping data recordings. From the rider’s point of view, it is preferable to have a visual, real-time output of rein tension values or show summary rein tension variables that can teach the rider to develop a better feel. An example is a device mounted on the headpiece of the bridle in which illumination of coloured LED lights indicates tension magnitude and left-right ratios. An alternative is to view the output on a smartphone mounted in a convenient location, that can process the data and display it graphically in various formats. 

## 4. Data Processing

### 4.1. Filtering

**Figure 7:**
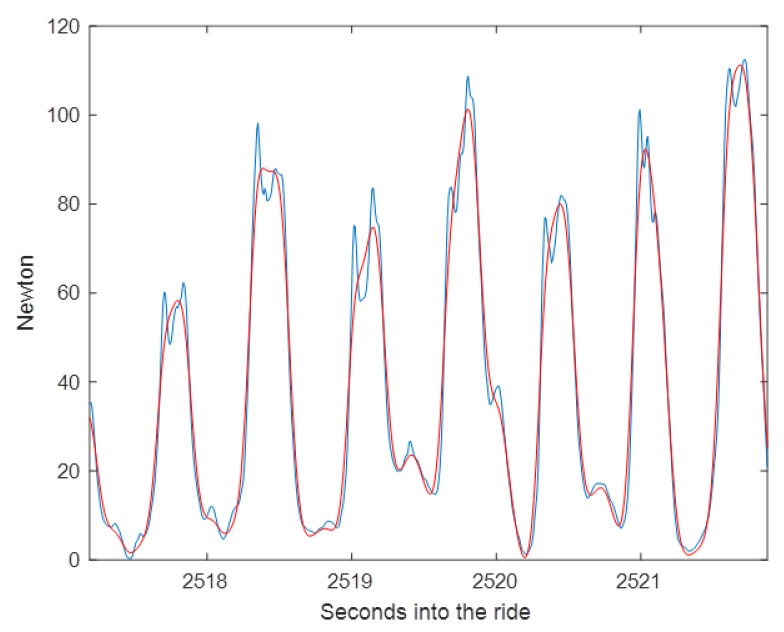
Rein tension data from the left rein in canter, sampled at 128 Hz. Original trace in blue and low-pass filtered trace in red (filter of order 2 and cutoff frequency 5 Hz). Note the loss and attenuation of the peaks.

Low-pass filtering, which passes signals with a frequency lower than the selected cutoff frequency and attenuates signals with higher frequencies, is usually not needed when studying rein tension. However, it may be useful to smooth the curve for visual purposes or to facilitate finding the targeted larger peaks when small peaks are abundant (Figure 7).

### 4.2. Stride Splitting 

Splitting the signal into strides facilitates identification of the repeatable patterns related to the gait cycle. In general, the goal is for stride splitting to be an automated procedure, but it may have to be manually supervised. Sometimes strides can be split directly from the rein tension data if the signal is sufficiently consistent. However, with a rider in a non-steady state situation, the rein tension signal tends to be chaotic, making it difficult to split strides based on repeatable, easily identified points/patterns. 

Stride splitting is best done by combining the rein tension signal with synchronized data from an independent source that allows easy recognition of stride events, such as synchronized video recordings, motion capture (mocap) measurements, or intertial measurement unit (IMU) data from one of the axial body segments or from the limbs. The croup has been found to produce a reliable signal for this purpose [22]. Data from the head are usually noisier compared to croup or limb movements [23].

### 4.3. Normalization (Time, Body Mass)

Time-normalization of individual strides is used to remove the effects of small differences in stride duration within an overall steady state. It facilitates superimposition of stride curves and generation of mean curves [8,9,13]. It may be valuable to view both time-normalized and non-normalized curves to fully understand the signal. Normalization to body mass is used frequently in biomechanical analysis to facilitate comparisons between horses of different sizes. With regard to rein tension, normalization to body mass has not been used and effects of horse size on rein tension measurements have not been reported. 

### 4.4. Synchronization with Other Devices

Synchronization of equipment is useful for understanding relationships between the rein tension data and other types of data, such as stride kinematics or electromyography. These studies call for precise synchronization of events. For other purposes, a less accurate manual synchronization may be sufficient to relate outside events to rein tension data.

Rein tension measurement systems rarely offer possibilities for hardware synchronization with other systems. An approximate synchronization can be accomplished manually, for example, by applying tension across the sensor while recording the movement with a video camera to provide synchronization with the kinematic measurements [12,13,24]. If using IMUs to log rein tension data as well as collecting kinematic data, the sensors can be synchronized by placing them together on a board which is tapped lightly while recording the events on video, then synchronizing the video with the rein sensors as described above [9]. The synchronization registrations are identified during data processing and the rein tension time vector is adjusted accordingly. Although there are inaccuracies in the manual synchronization methods, it may be adequate to (statistically) relate rein tension events with locomotor events, especially if a sufficient number of strides have been collected in several horses. When rein tension data can be viewed next to accelerometer data (e.g., from the poll, croup or limbs) it is possible to verify that synchronization looks reasonable based on information from previous studies. Simultaneous kinematic data collection allows automatic segmentation of the ride into gaits and tracks/exercises (straight/circle), together with details of the movements of horse and rider. 

## 5. Rein Tension Variables

**Figure 8:**
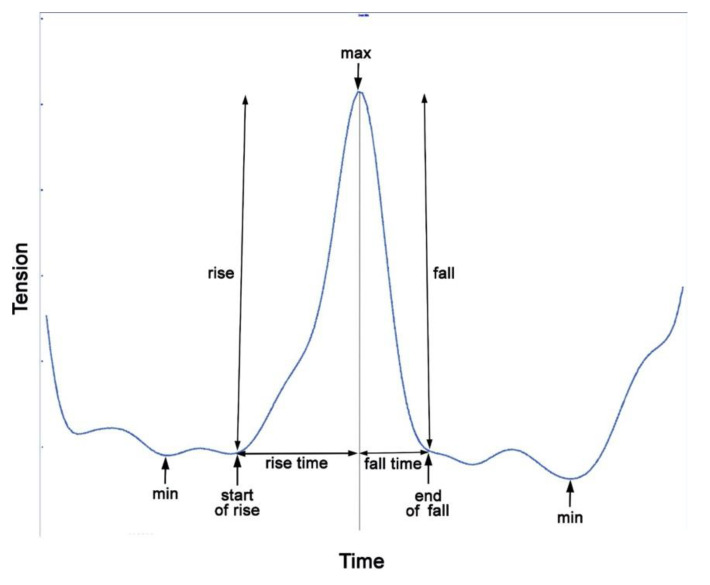
A single rein tension spike from a trotting horse with additional data at the start and end of the spike. The discrete variables extracted and calculated from the data are indicated. A typical single rein tension spike starts at the inflexion where tension begins to rise rapidly to a maximum, after which it decreases rapidly until the trace makes an inflexion at the end of the rapid decrease. An initial minimum (start of rise), a maximum and a final minimum (end of fall) value can be measured. This pattern is still more or less obvious when other (smaller and larger) peaks are superimposed.

In order to quantify and analyze rein tension, discrete values, such as minima and maxima, are extracted from the time-continuous signal, most often on a per stride basis. Figure 8 shows how the variables are extracted.

### 5.1. Temporal Variables

The main temporal variables relate to the timing of the minima and maxima of the rein tension spikes. From these time points, spike durations, rise time and fall time are calculated. Timing variables may be calculated from the original data or from time-normalized stride data as appropriate for the objectives of the study. 

### 5.2. Maxima and Minima

During steady state movement, successive rein tension minima and maxima occur at fairly consistent intervals and this can be used as one of the selection criteria for locating the values and detecting when a spike is absent. The values can be extracted manually or an algorithm can be written to automate the process based on the repetition frequency of the events. The process is facilitated if the data are first split into strides based on an external kinematic reference, e.g., limb ground contact times from video [4,5], mocap [25] or IMU data [26]. For segmented strides, the time of occurrence of the minima and maxima can be extracted with or without time normalization. 

Rein tension is greatly influenced by gait, but also by the style of riding: for example, in canter, the magnitude of rein tension is lower when adopting a two-point position as opposed to a seated position [12,27]. When riding ‘on a contact’, the rider keeps the reins sufficiently short to maintain a baseline level of tension which is in contrast to riding without contact, when the reins have a loop between the hand and the bit. However, even when riding without contact, there are small, rhythmic changes in rein tension due to the inertial effects of the vertical movements of the horse’s head, neck and body which cause the reins to bounce at the horse’s stride frequency. 

When riding on a contact, minimal (or baseline) tension may be zero or greater than zero; it varies with gait, horse and rider technique. The minimal tension represents what the rider perceives as their ‘contact’ with the horse [6]. Higher minimal values are present in horses that feel heavier in the rider’s hand. 

Maximal and minimal tension values showed the best ability to discriminate between side reins with different levels of compliance, though changes in the minimal and maximal values were in opposite directions, indicating that they provided different types of information. For example, a more compliant rein had higher minima and lower maxima compared with a less compliant rein [6]. Maximal tension is influenced by mechanical oscillations of the horse’s head and neck by the superimposed effects of the rider’s aids, or by voluntary head-neck movements by the horse [23]. These values may be influenced by a rein aid or an intentional release of the rein by the rider, e.g., during a half halt. In trot and canter a balancing half halt should be given just before or around the time when nose down rotation begins. Thus, rein tension peaks in the suspension phase of cantering horses, at a time when values are inherently low, have been interpreted as an effect of the rider’s rein aids. The aids change not only the magnitude, but also the timing of the rein tension peaks [24].

**Figure 9:**
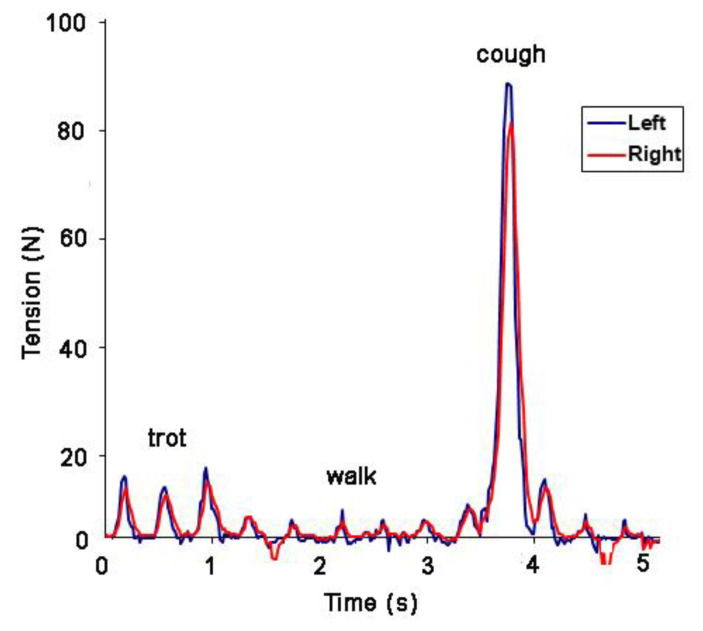
Rein tension data for a horse that coughed during data collection at walk.

Based on the undulant nature of the rein tension pattern, it has become apparent that measurement of a single maximal value from a data string (e.g., [8]) as the sole variable is of limited value in analyzing rein tension, especially if the data include an atypical event such as the horse stumbling, coughing or rooting on the reins (Figure 9). It is recommended that rein tension values be extracted on a stride-by-stride basis, from a sufficient number of strides for statistical analysis. If video or mocap data are available, strides that contain atypical events can be removed.

### 5.3. Spike Magnitude and Rate of Tension Change

Spike magnitude is the difference between the initial minimum at the start of the rise and the next maximum and is calculated by subtraction (Figure 8). It is influenced by extrinsic events in a similar way to the maximum value. Spike magnitude is related to the amount of movement of the head and neck [6] and could, therefore, be influenced by conformation. 

To quantify how quickly the rein tension changes, the rate of rise and fall can be calculated. The rise is the change in tension between the start of the rise, which often does not coincide with the minimal value, and the maximal tension. Rise time is the time over which the rise occurs. Rise rate is calculated by dividing the magnitude of the rise by the rise time (Figure 8). 

The fall is the difference between the maximal value and the value at the end of the fall. Fall time is the time over which the decrease in tension occurs. Fall rate is calculated by dividing the fall magnitude by the fall time. The rates of rise and fall are influenced by rein length and compliance [6] and the timing of the rider’s aids (half halts). 

### 5.4. Measures of Central Tendency

The mean and median are measures of central tendency based on tension values during the entire stride. The distribution of rein tension time-series data is usually highly skewed with the majority of values being clustered towards the low end of the range and/or the presence of a low number of exceptionally high values. For normally distributed data the mean and median are almost the same, but when the distribution is highly skewed, as with rein tension data, the mean value does not provide a good representation of the average, and the median gives a better representation of central tendency. Despite the fact that the mean has limited value for understanding the pattern or magnitude of rein tension, it has been the only variable reported in several studies [28,29,30]. It may be advisable to report both mean and median values to demonstrate skewness and to facilitate comparisons with previous studies.

### 5.5. Impulse

Impulse is a useful summary variable that represents the total amount of tension applied over a specified period of time, which might be a single spike, a full stride or several strides. The value depends on both the magnitude of the force and the time over which the force is applied. Impulse is measured as the area under the curve and is calculated mathematically by time integration of the rein tension curve in units of Newton-seconds (Ns). 

## 6. Statistical Considerations

### 6.1. Required Number of Horses, Riders, Strides

As in other research areas, power calculations should guide the choice of sample size when designing a study. Both horses [12] and riders [15] make large contributions to variation in the rein tension signal. The required numbers of horses and riders should be determined according to the objectives of the study [31,32,33], as it may limit the possibility for extrapolation to the general population, when the substantial variation between individual horses and riders is considered. If studying subtle aspects of the rein tension pattern, e.g., left-right rein tension differences or rein aids, a more substantial sample size may be needed than for studying gait-related differences since these per se are large. In relation to lameness, it has been suggested that ~20–25 strides are required to evaluate vertical movement asymmetry. The rein tension signal tends to be more variable than the vertical displacement of the body, so the number of strides needed to evaluate mean or median rein tension, for example, is likely to be at the upper end or above that range. 

### 6.2. Determining Outliers

Outliers may be produced by haphazard or erratic actions by the horse, and the context of occurrence will guide whether these should be removed or not. Synchronized video recordings of the experiment are very helpful for making these decisions. The overall necessity of removing outliers depends on their frequency and the statistical methods chosen. Analyzing data before and after outlier removal and comparing the results can inform the decision further. If outlier removal is deemed necessary, it has been suggested to remove data points as outliers if their value exceeds 1.5 SD from the mean. 

### 6.3. Multivariable Statistical Analysis

Multivariable methods, such as linear mixed effects regression, are often the method of choice for analysis of biomechanical variables. Mixed models have been used to analyze discrete descriptive variables such as median or maximum rein tension [12]. A random part is needed in analyses of stride-by-stride data, since multiple measurements are made on the same individual (several strides), and often two reins at the same time. The ‘chaotic’ variation in rein tension between strides suggests that this variation should be taken into account during the analysis and not hidden in trial or horse averages. The random effects can control for repeated observations on multiple levels, for example riders, horses or side (left/right) within horse. The choice of random effects structure can sometimes require statistical advice. One issue when using mixed models is that rein tension data most often need to be square root transformed in order to conform with the requirement for normal distribution of residuals. Analysis of transformed data, when needed, yields correct p-values, while the estimates (or least square means) are calculated on the transformed scale and back-transformed figures are only approximately correct. It must also be remembered that a relationship that is linear on the transformed scale is nonlinear on the original scale. Results from models on transformed data should therefore also be reported on the transformed scale. 

### 6.4. Time Series Analysis

In addition to analyzing discrete values, it is also possible to apply various forms of time series analysis. One approach is to use the time index of a normalized stride (e.g., 0–100%) as a fixed factor in a mixed model, producing estimated stride curves with the possibilities for significance testing [12]. Another approach is to use generalized adaptive modeling (GAM) technique to create a smooth estimate of the rein tension time series, which can be applied on a stride basis or even over whole riding sessions [15].

### 6.5. Left-Right Rein Comparisons

Bilateral transducers can quantify differences between the left and right reins on straight lines and between the inside and outside reins when moving through a turn, on a circle or performing lateral movements. Coordination between the left and right reins becomes evident when bilateral rein data are available. If rein tension is measured bilaterally, it allows evaluation of left-right asymmetries associated with handedness of the rider or sidedness of the horse, though it cannot differentiate between the two. Asymmetries of the rider and horse probably also interact, making them even more difficult to disentangle, which should be taken into account during the analysis. Most studies report results for left and right reins separately and left-right differences are usually relatively small in comparison to differences between gaits, riders or horses. When left-right comparisons are of interest, the traces should be well matched with other extraneous effects removed (i.e., a well-controlled experimental situation) or use linear mixed models with appropriate fixed and random effects where such factors can be controlled. 

When left-right differences are not of interest, data can usually be pooled. In mixed models, this can be handled by simply adding an extra term (side within horse) to the random structure. Other alternatives are to use the mean or sum of the left and right rein values, based on the fact that both reins act simultaneously in creating bit pressure in the horse’s mouth. 

## 7. Recommendations for Reporting Rein Tension Studies

In order to standardize reporting of data and facilitate comparisons between publications, it is recommended that, in accordance with the objectives of the study, relevant items from the following list be included in rein tension manuscripts:

- Horses: number, level of training. If available: age, height, weight, dental history, information regarding side preference;
- Bridle type and fit, including noseband type and adjustment;
- Bit type;
- Reins: material, length and adjustment of side reins;
- Riders: number, weight, height, level of equestrian experience, handedness (preferably based on multiple tasks);
- Style of riding: loose reins or on a contact, seating style (sitting, rising, light seat), position/balance;
- Sensors: type of transducer, model and manufacturer, weight, specifications regarding sensitivity and range, frequency and method of calibration, sampling frequency, transmission and storage of data;
- Data recording conditions: surface/footing, indoors/outdoors, gait, speed, direction (straight, turn left, turn right), turn/circle radius and relevant environmental details (weather and surroundings);
- Data analysis: filtering method and parameters, stride splitting method, extraction of discrete data points, calculation of variables, statistical analysis.

## 8. Conclusions

Given the direct connection from the rider’s hand to the horse’s mouth, rein tension data can have welfare implications since the application of high forces to the horse’s mouth may cause pain, discomfort, and oral lesions. An important application of rein tension measurements lies in their use as a tool to improve equestrianism and horse welfare. This requires the collection of accurate data, calculation of appropriate variables, and the use of valid analytical methods. The optimal choices may vary depending on the purpose of the measurements.

Although rein tension devices are becoming widely used, there is a paucity of guidance and standardization regarding the technical requirements of these devices and how to treat the resulting data. This paper provides information on how to select and interface the components of a rein tension measurement system to facilitate collection of accurate and reliable data. It also offers guidelines for the analysis and interpretation of rein tension variables. The main recommendations are to choose sensors with an appropriate range and sensitivity based on existing literature; calibrate sensors before and after each data collection (and during data collection if recommended by the manufacturers); analyze variables that are appropriate to the study objectives; and check the statistical distribution of the variables and transform if necessary.
